# Human Term Placental Cells: Phenotype, Properties and New Avenues in Regenerative Medicine

**Published:** 2012

**Authors:** Maddalena Caruso, Marco Evangelista, Ornella Parolini

**Affiliations:** Centro di Ricerca E. Menni, Fondazione Poliambulanza, Istituto Ospedaliero, Via Bissolati 57, 25124 Brescia, Italy.

**Keywords:** Amnion, chorion, placenta, regenerative medicine, stem cells

## Abstract

The human placenta has long been the subject of scientific interest due to the important roles which it performs during pregnancy in sustaining the fetus and maintaining fetomaternal tolerance. More recently, however, researchers have begun to investigate the possibility that the placenta’s utility may extend beyond fetal development to act as a source of cells with clinically relevant properties. Indeed, several groups have reported the isolation of cells from different placental regions which display both multilineage differentiation potential and immunomodulatory properties *in vitro*. Furthermore, these cells have also been shown to secrete soluble factors involved in pathophysiological processes that may aid tissue repair. Cells with such features will clearly find application in the field of regenerative medicine for the repair/regeneration of damaged or diseased tissues or organs.

In line with these promising findings, several preclinical and clinical studies conducted to date argue in strong favor of the therapeutic utility of placenta-derived cells for the treatment of several diseases. Although much work remains to be conducted in order to fully understand the properties of placental cells and the mechanisms which underlie their beneficial effects *in vivo*, data reported to date nonetheless provide compelling evidence in support of the placenta as a cell source for use in regenerative medicine.

The placenta is a temporary fetomaternal organ that is formed during pregnancy, acting as an interface between mother and fetus to facilitate important functions such as nutrition, protection, respiration and excretion. Another important role of this organ is the maintenance of fetomaternal tolerance, which represents a fascinating yet still not entirely understood phenomenon that is considered to be the best known example of immune tolerance, considering that it allows the maternal immune system to tolerate the genetically distinct fetus.

For many years, the study of placental structure, development and occasional abnormalities has provided important insights into fetal development, as well as into fetal dysfunction and pregnancy complications. Recently, however, scientific interest in this organ has increased even further due to mounting evidence which implicates it as an appealing material for research due to its potential clinical applications. Indeed, placental tissues harbor stem/progenitor cells with properties which make them attractive candidates for application in regenerative and reparative medicine (1-3). Moreover, considering that the placenta is normally discarded after birth as medical waste, its procurement as a cell source is easy and raises no ethical controversy. Progress in our understanding of the biology and properties of placenta-derived cells has encouraged researchers to investigate their effects in animal models of different diseases, with the ultimate aim of developing clinical applications based on the use of these cells. 

Here, beginning with an overview of the structure of the human term placenta, we will then provide a description of the different stem/progenitor cell types that have been studied from different human placental tissues, in terms of their isolation methods, morphology, phenotype, differentiation potential and immunomodulatory properties. We will also report on the encouraging results which have been described to date through the application of these cells in preclinical and clinical settings.

**Structure of the human placenta**

The human term placenta is a discoid-shaped organ which is 15–20 cm in diameter and 2–3 cm in thickness. It is composed of both a fetal component and a maternal component. 

The fetal component is composed of all placental portions which originate from the blastocyst, including the placental disc, the amniotic and chorionic membranes (often referred to as the fetal membranes) and the umbilical cord. The maternal component is termed the decidua, and is derived from the maternal endometrium. Beginning from the fetal side (Fig. 1A), the innermost portion of placenta is the amniotic membrane (also termed amnion) (Fig. 2A). The inner surface of the amnion is in direct contact with the amniotic fluid, and encloses the fetus in the amniotic cavity (sac). The amnion is an avascular tissue composed primarily of two cell types, namely, the epithelial cuboid cells and the columnar cells. It is the amniotic epithelial cells which form the continuous lining that is in direct contact with the amniotic fluid (Fig. 2A). 

The umbilical cord extends from the amnion toward the fetus (Fig. 1A). Normally, it contains two umbilical arteries and one umbilical vein (Fig. 2C). These are embedded within a gelatinous, proteoglycan-rich matrix known as Wharton’s jelly, which prevents the compression, torsion and bending of the umbilical vessels (Fig. 2C).

On the other side of the amniotic epithelium lies a thin layer of amniotic mesoderm (Fig. 2A), which contains an acellular compact layer of interstitial collagens I, III, and fibronectin, and a deeper network of widely dispersed fibroblast-like mesenchymal cells and rare macrophages. The amniotic mesoderm is in turn connected to the placental disc (Fig. 1 A-C and Fig. 3) which is composed of the chorionic plate and the basal plate, that together form a base and cover, respectively, to enclose the intervillous space (described below) (Fig. 3). 

The chorionic plate is multilayered, and consists of a spongy layer followed by the chorionic mesodermal layer, and a layer of Langans’ fibrinoid which is interposed with highly variable amounts of proliferating extravillous cytotrophoblast cells. The spongy layer loosely connects the amniotic mesoderm and the chorionic mesoderm. The basal plate, meanwhile, forms an intimate and important contact zone between maternal and fetal tissue. Protrusions extending from the basal plate into the intervillous space produce the placental septa, which divide the fetal part of the placenta into the irregular cotyledons (Fig. 3). 

The region between the chorionic plate and the basal plate contains the chorionic villi**,** which project from the chorionic plate and either anchor the placenta through the basal plate to the endometrium, or terminate freely in the intervillous space, where maternal blood flows (Fig. 3). 

**Fig 1:**
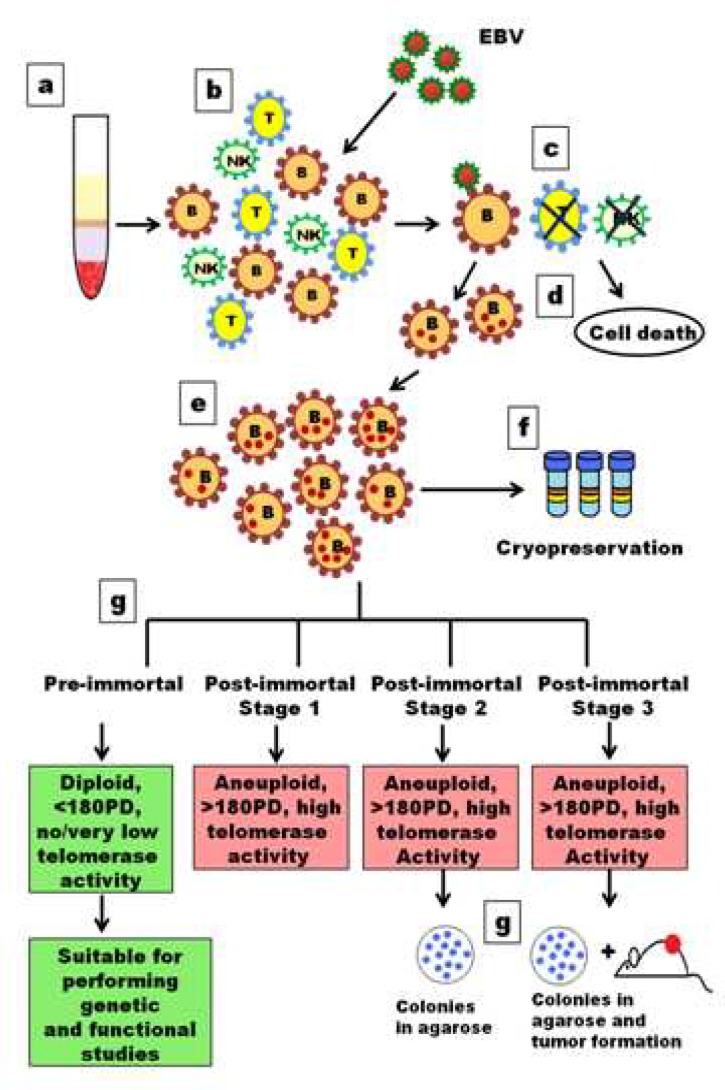
Human term placenta. Panels A, B and C show the placenta from the fetal, maternal and lateral sides, respectively

**Fig 2:**
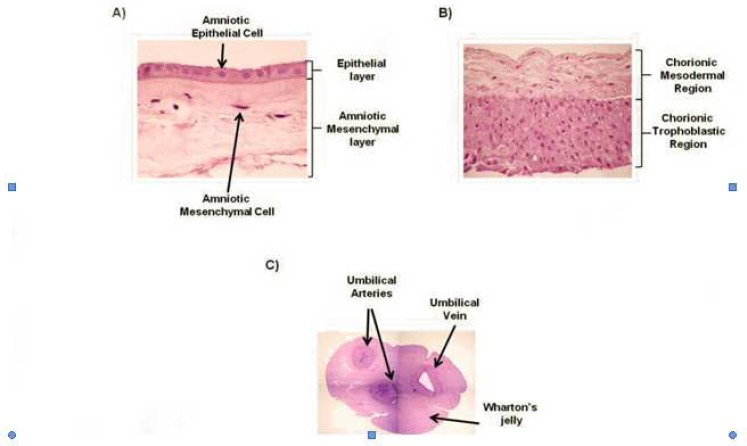
Cross-sectional representation of the human amniotic membrane (A), chorionic membrane (B) and umbilical cord (C)

**Fig 3:**
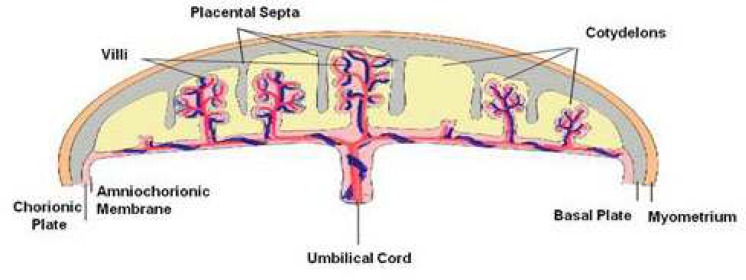
The figure shows a schematic representation of the different regions of the placental disc

This results in intimate contact between the fetal and maternal circulation, allowing fetal blood which has been transported to the chorionic villi via the branches of the umbilical arteries to absorb oxygen and nutrients from, and transfer waste products to the maternal blood through the villous walls, before being returned to the fetus through the umbilical vein. 

Beyond the margins of the chorionic plate, the intervillous space is obliterated, resulting in fusion of the chorionic plate and the basal plate to form the chorionic membrane (commonly called the chorion leave), made up of a chorionic mesodermal and chorionic trophoblastic region (Fig. 2B). The amniotic and chorionic membranes together extend from the edges of the chorionic plate to enclose the fetus in the amniotic cavity. Meanwhile, the maternal portion of the placenta (deciduas) serves to anchor the placenta to the endometrium during gestation.

**Placenta-derived stem/progenitor cells**

The fact that placental tissues begin to form during the first stages of embryological development has prompted scientists to explore the possibility that these tissues may harbor cells which have retained some of the stemness that is characteristic of the early embryonic cells from which they derive. Meanwhile, the fact that the placenta plays a fundamental role in fetomaternal tolerance suggests that cells derived from placental tissues may also have immunomodulatory properties. These two key features therefore make the placenta an excellent candidate cell source for use in an allo-transplantation setting in regenerative medicine, with the possibility of providing cells which can differentiate into many different cell types as well as exerting immunomodulatory effects. 

In accordance with this hypothesis, an ever-increasing number of studies reports the isolation of several cell types with stem/progenitor characteristics from placental tissues. Examples include human amniotic epithelial cells (hAECs), which are isolated from the amniotic membrane (1, 4), as well as cells with characteristics of multipotent mesenchymal stromal cells (MSCs), which have been isolated from both fetal and maternal placental tissues (1, 5-6).

hAECs are cuboidal to columnar cells which form a monolayer that is in direct contact with the amniotic fluid (Fig. 2A). These cells are efficiently recovered after separation of the amniotic membrane from the chorionic membrane, followed by digestion of amniotic membrane pieces with trypsin either before (7) or after (8) the removal of amniotic MSCs. Generally, a term AM yields between 80 to 300 × 10^6^ hAECs (7). These cells can usually be maintained in culture for 2–6 passages before proliferation ceases (1) and the cells begin to undergo morphological changes whereby they adopt an elongated stromal-like appearance, whilst also beginning to express markers suggestive of an epithelial to mesenchymal transition and showing reduced differentiation potential (9-10).

To date, MSCs have been isolated from several placental regions, namely *i)* from the fetal membranes, which yield human amniotic mesenchymal stromal cells (hAMSCs) and human chorionic mesenchymal stromal cells (hCMSCs) (1, 6, 8, 11); *ii)* from the chorionic villous stroma of first-trimester placenta (12); *iii) *from at least five compartments of the umbilical cord, namely, the umbilical cord blood, the umbilical vein subendothelium, and three regions of the Wharton’s jelly: the perivascular zone, the intervascular zone, and the subamnion (5)*; iv) *from different regions of the decidua (6), and *v)* from the fetal chorionic villi of term placenta (6, 13-14), although it should be noted that isolation of cells from this region carries a risk of contamination with maternal decidual cells (16), and it is therefore important to confirm the fetal origin of cells isolated from this region using methods which are sensitive enough to detect levels of maternal cells of less than 1% (1). Meanwhile, some studies also report the isolation of MSCs from mixed portions of placenta which include more than one tissue (17-19).

In general, placental MSCs exhibit plastic adherence and fibroblast-like morphology in culture. They can be isolated through various methods, which are generally based on digestion of the placental pieces of interest with various concentrations and incubation times of different enzymes, including dispase, collagenase and DNase (1, 8, 12, 20). In some cases, as for chorionic villi-derived MSCs, the isolation method may involve mechanical mincing of the chorionic tissue followed by trypsin digestion and seeding in a medium which allows colony formation and culture of fibroblast-like cells (15), although the explant culture method (13), whereby cells are outgrown from pieces of chorionic villi attached to dishes, has also been successfully applied. Explant methods are also used for the isolation of Wharton’s jelly-MSCs (21-22). In terms of yield, for example it has been reported that 20-50 X 10^6^ hAMSCs can be obtained from one placenta at term (3, 23). Both hAMSCs and hCMSCs adhere and proliferate on tissue culture plastic, and can be maintained in culture until passage 5–10 (1). Furthermore, hAMSCs have been shown to be clonogenic, forming colonies that could be expanded for at least 15 passages (8, 24).

**Placental cell phenotype**

Both hAECs and placental MSCs express several cell surface and intracellular markers typical of stem/progenitor cells, such as Stage Specific Embryonic Antigen (SSEA)-4, Tumor Rejection Antigen (TRA)1-60, TRA1-81, and octamer-binding protein (OCT)-4 (1, 3, 22, 25).

In accordance with the basic criteria that are referred to for defining MSCs, as set out by the International Society for Cellular Therapy (26), placental MSCs are positive for CD73, CD90 and CD105, while they do not express CD45, CD34, CD14 and HLA class II (1, 5, 12, 25, 27). 

For some placental cells, such as amniotic membrane-derived cells, the isolation protocol used may influence the levels and pattern of marker expression, and such expression patterns may also vary with passage number (28). For instance, Murphy et al. have recently reported changes in the expression levels of a selection of cell surface markers in hAECs between P0 and P5 (29). Meanwhile, changes in marker expression have also been observed with varying gestational age (12, 30). 

Interestingly, placental cells have also been reported to express several lineage-associated genes, suggesting that they could act as progenitors and differentiate into various cell types. Further details regarding the expression of such genes in hAECs and hAMSCs can be found in the review of Manuelpillai et al. (3).

**In vitro** **multilineage differentiation capacity of placental stem/progenitor cells**

When appropriately stimulated during *in vitro* culture, placental stem/progenitor cells are able to undergo multilineage differentiation, and even have the capacity to differentiate across germinal boundaries outside of their specific lineage (1). For a detailed description of hAEC differentiation in vitro, we refer the reader to the review of Manuelpillai et al. (3). When considering differentiation of hAECs, it is also important to note that culture and expansion conditions may influence the differentiation capacity of these cells. For example, Pratama et al. (31) have recently shown that serial expansion of hAECs in xenobiotic-free media hampers their differentiation towards chondrocyte, hepatocyte, α-and β-pancreatic-like cells. 

As for other MSCs, placenta-derived MSCs are able to undergo differentiation toward the osteogenic, chondrogenic, and adipogenic lineages (1, 5, 12, 15). Furthermore, they are also able to differentiate toward several other lineages, including neurogenic (1, 3, 5, 12, 15), cardiomyogenic (3), myogenic (1, 3, 12, 15, 32), angiogenic (1, 3, 33); and pancreatic (1, 34). A recent study has also shown that Wharton’s jelly-derived MSCs are able to undergo *in vitro* differentiation into germ-like cells (35).

**Immunomodulatory properties of placental stem/progenitor cells**

Cells from different placental regions, like human bone marrow-derived MSCs, are poor antigen-presenting cells, due to the fact that they express low levels of the MHC class II and co-stimulatory molecules (1).

Through in vitro studies, our group has shown that amniotic and chorionic MSCs fail to induce an allogenic or xenogenic immune response in mixed lymphocyte reactions (11). Furthermore, we have also shown that human amniotic membrane-derived MSCs do not elicit an allogenic T-cell response, and also inhibit lymphocyte proliferation which has been induced by allo-antigens or T cell receptor cross-linking. The mechanism of such inhibition is likely to be based on the action of soluble factors secreted by the cells (36). In addition, these cells have also been shown to inhibit the generation and maturation of monocyte-derived dendritic cells, through arrest of the cells in G0 phase of the cell cycle as well as abolition of the production of some inflammatory cytokines (37). In keeping with these results, other groups have also shown that placental MSCs exert immunosuppressive effects, often in a dose-dependent manner (38-41).

It is worth noting that the immuno-modulatory properties of placental cells may also be influenced by the conditions under which they are cultured and expanded. For example, failure to remove the HLA-DR^+^ subpopulation before expansion of amniotic mesenchymal cells (36) may partially abrogate their immunosuppressive effects *in vitro or in vivo*.

Placental cells have also been shown to be capable of successful engraftment and long-term survival in various organs and tissues after in utero transplantation into pregnant rats (42), as well as following transplantation into neonatal animals (11). Notably, organs of transplanted animals have been shown to display human microchimerism without evidence of inflammation or rejection, therefore indicating active tolerance of the cells (11). 

**Preclinical and clinical studies with placental stem/progenitor cells**

The properties displayed by placental stem/progenitor cells have led scientists to seek to take advantage of these by studying their therapeutic potential in animal models of different diseases. 

In this regard, successful results have been reported for the treatment of neurological disorders, including Parkinson’s disease (PD), stroke and spinal cord injury (SCI). Indeed, transplantation of both hAECs and Wharton’s jelly-derived MSCs in rodent models of PD has been shown to improve neurobehavioral deficit and promote preservation/recovery of dopaminergic (DA) neurons (43-46). Furthermore, transplantation of hAECs and MSCs isolated from decidua has been shown to result in beneficial effects in rats with stroke (47-48), resulting in a significant improvement in behavioral dysfunction and a reduction of infarct volume. Meanwhile, rat models of SCI have been used to show that transplantation of hAECs (49) and Wharton’s jelly-derived MSCs (previously differentiated into neurospheres in vitro) (50-51) are able to promote functional recovery.

Pancreatic diseases represent another area where preclinical studies have shown the therapeutic value of placental MSCs and hAECs (34, 52-54). Notably, a pilot phase I clinical trial which was recently performed by Jiang et al. (17) demonstrated the beneficial effects of placental MSCs prepared according to GMP guidelines for the treatment of patients with type 2 diabetes. 

Treatment based on the use of placental cells has also been investigated for fibrosis affecting major organs, such as lung and liver. Our group (55) has demonstrated that both allogeneic (murine cells into mice) and xenogeneic (a mix of hAECs, hAMSCs and hCMSCs into mice) transplantation of fetal membrane-derived cells significantly reduced the severity of lung fibrosis when administered by various routes of delivery into immunocompetent mice with bleomycin-induced lung injury. Similarly, Moodley et al. (56, 57) have reported beneficial effects after transplantation of Wharton’s jelly-derived MSCs and hAECs into bleomycin-challenged immunocompromised (SCID) mice. Meanwhile, animals with carbon tetrachloride (CCl4)-induced liver injury have been used to demonstrate the feasibility of placental cell-based therapies for reducing liver fibrosis (58-60). 

Placental cells also show promise for the treatment of peripheral vascular diseases (61). Recently, data from clinical trials have shown that cell therapy using human placental cells which have been expanded in a 3D bioreactor is safe and results in improved quality of life and efficacy measurements in patients with critical limb ischemia (http://www.pluristem.com). 

The use of placental cells has also been investigated for repair/regeneration of cartilage and bone defects *in vivo*. To this end, reparative effects have been observed in nude rats with cartilage defects after transplantation of chorionic villi-derived MSCs (62) and hAMSCs (63), which were generally implanted after being embedded in a collagen scaffold. Meanwhile, other authors have proven the efficacy of cells isolated from a chorionic MSC cell line in facilitating bone repair in a bone fracture mouse model (64).

Although the mechanisms underlying the therapeutic effects of placental cells which have been observed *in vivo *remain to be elucidated, the majority of reports in current literature seem to suggest that these effects are more likely to be due to paracrine actions performed by bioactive molecules secreted by the placental cells, rather than their ability to differentiate toward a specific lineage. Indeed, it has been shown that placenta-derived cells can secrete several factors which are involved in various pathophysiological events, such as cytokines with immunomodulatory and anti-inflammatory properties (65), angiogenic factors which promote wound healing (65-66), growth factors associated with cell proliferation and differentiation (43, 65-68), and anti-apoptotic and anti-oxidative factors (69). It is therefore plausible that transplanted placental cells may exert their beneficial effects by acting on surrounding host cells and the microenvironment which they occupy, in order to promote endogenous tissue repair or to elicit the beneficial effects in the host (anti-inflammatory, anti-scarring, angiogenic, etc.) 

## Conclusions

Here we have provided an overview of the interesting characteristics possessed by placental cells which make them promising candidates for use in regenerative medicine. In particular, their *in vitro *multilineage differentiation potential, as well as their absent or low immunogenicity and immunomodulatory properties, together support their utility in regenerative medicine, particularly in allogeneic transplantation settings and for diseases where immunomodulatory properties may have therapeutic effects (*i.e.* inflammatory and fibrotic diseases). These cells are also able to secrete a number of factors involved in various pathophysiological events with potential therapeutic effects, and several experiments in preclinical and clinical settings have now proven their therapeutic value for different pathological conditions. Moreover, the fact that placental tissue is normally discarded after birth and is therefore available in plentiful supply, while its procurement is safe and non-invasive, together with the fact that its use raises no ethical controversy, means that the placenta represents an extremely attractive source of cells for research and therapeutic purposes. 

Although much work has already been carried out in defining the characteristics and properties of placental cells and assessing their applicability for the treatment of various diseases, further extensive studies of these cells are still required. In particular, isolation methods which yield more homogeneous cell populations, as well as a better understanding of the precise location within the placental tissues from which each different cell type derives, and a more extensive knowledge of their phenotype and stem cell potential, are all required in order to address gaps in our current understanding of these cells. Furthermore, the question as to whether the beneficial effects of these cells in reducing inflammation and promoting repair/regeneration are due to differentiation of the cells themselves or to paracrine actions which they exert on the surrounding host tissue also remains to be fully addressed, even though current evidence tends to support the latter of these hypotheses. These key pieces of information, together with demonstration of the safety and efficacy of placental cell transplantation, will no doubt drive forward the successful development of therapeutic treatments based on the use of these cells.
